# The Promising Potential of *Caulerpa microphysa* in Dermatology

**DOI:** 10.3390/cimb46070452

**Published:** 2024-07-18

**Authors:** Chang-Hsun Ho, Chan-Yen Kuo

**Affiliations:** 1 Department of Anesthesiology, Show Chwan Memorial Hospital, Changhua 500, Taiwan; jikeo2003@gmail.com; 2 Department of Research, Taipei Tzu Chi Hospital, Buddhist Tzu Chi Medical Foundation, New Taipei City 23142, Taiwan

In recent years, the search for natural compounds with therapeutic properties has gained momentum, with marine organisms emerging as rich sources of bioactive substances [1]. Among these, seaweeds have shown remarkable promise due to their unique chemical composition and adaptive mechanisms [2]. Lu et al., in a study published in *Current Issues in Molecular Biology*, presented compelling evidence of the beneficial effects of *Caulerpa microphysa*, a green alga, in promoting collagen homeostasis and inhibiting inflammation in skin cells [3].

*Caulerpa microphysa* has been used to explore the potential of polysaccharides in cosmetic products. The green alga, native to Taiwan, shows promise owing to its high polysaccharide content, which comprises 73.93% of its extract. These polysaccharides mainly consist of mannose, glucose, and galactose. Additionally, the authors also suggested that *Caulerpa microphysa* polysaccharide extract (CME) could be a valuable ingredient in cosmetic formulations, offering natural and effective benefits for skincare [4]. Moreover, the pepsin-digested extracts of *Caulerpa microphysa* were tested in vitro for their anti-tumor effects in BALB/c mice transplanted with myelomonocytic leukemia (WEHI-3) and human promyelocytic leukemia (HL-60) cell lines. The extracts inhibited the growth of both cell lines and induced DNA damage [5]. A study conducted by Lu et al. highlighted the superior anti-inflammatory and collagen-stabilizing properties of the ethyl acetate (EA) extract of *Caulerpa microphysa* compared with those of its water extract. This non-water-soluble extract led to a significant reduction in pro-inflammatory cytokines (IL-1β, IL-6, IL-8, and TNF-α) and enhanced the expression of type I procollagen and collagen in human fibroblasts. These findings suggest that *Caulerpa microphysa* is a valuable natural ingredient for dermatological applications, particularly in anti-aging and anti-inflammatory skincare products [3]. Furthermore, *Caulerpa microphysa* extracts may have potential anti-tumor and anti-inflammatory effects, warranting further investigation for their use as health foods.

The anti-inflammatory and collagen homeostasis effects of the EA extract are attributed to the presence of four key terpenoids: siphonaxanthin, caulerpenyne, caulerpal A, and caulerpal B. These compounds were identified using advanced UHPLC-QTOF-MS analysis, underscoring the importance of modern analytical techniques in natural product research [3]. However, the effects of caulerpal A and caulerpal B on dermatology are limited.

Siphonaxanthin, a safe dietary carotenoid, could help reduce the risk of atherosclerosis by mitigating advanced glycation end product (AGE)-induced inflammation through endoplasmic reticulum (ER) stress and nuclear factor-κB (NF-κB) pathways [6]. Additionally, siphonaxanthin has been shown to markedly inhibit the viability of human leukemia HL-60 cells by inducing apoptosis, outperforming fucoxanthin in reducing cell viability, and exhibiting higher cellular uptake [7]. Siphonaxanthin demonstrated notable antiangiogenic activity by downregulating signal transduction via fibroblast growth factor receptor-1 in vascular endothelial cells [8]. These promising findings suggest the need for further research, particularly in vivo studies, to validate the bioactive effects of siphonaxanthin and determine its bioavailability and metabolic fate [9].

Caulerpenyne (Cyn), a toxin derived from the seaweed *Caulerpa taxifolia*, inhibits after-hyperpolarization in invertebrate neurons [10]. Parent-Massin et al. reported that the cytotoxic effects of caulerpenyne in various in vitro models, including skin cells, primary melanocyte and keratinocyte cultures, immortalized keratinocytes (HaCaT and HESV), and bone marrow cells (hematopoietic progenitor CFU-GM). Additionally, the concentrations of caulerpenyne necessary to induce cellular toxicity suggest that the risk of cutaneous or food intoxication in humans is minimal [11].

Although Lu et al. provided robust evidence, their study also opened avenues for further research. Future studies should focus on elucidating the precise mechanisms through which these terpenoids exert their effects at the molecular level. Clinical trials are essential to confirm the efficacy and safety of *Caulerpa microphysa* extracts in humans [3].

This communication underscores the therapeutic potential of *Caulerpa microphysa*, a green alga rich in bioactive polysaccharides and terpenoids. Previous findings have highlighted this alga’s anti-inflammatory, anti-tumor, and collagen-stabilizing properties, suggesting its utility in dermatological applications and health foods. Key compounds, such as siphonaxanthin and caulerpenyne, were identified as critical to these effects, demonstrating important anti-angiogenic and apoptosis-inducing activities. Despite the promising in vitro results, further in vivo studies and clinical trials are essential to fully understand the mechanisms, bioavailability, and safety of these compounds in humans. This research sets a solid foundation for the future exploration of *Caulerpa microphysa* in natural-product-based therapeutics and cosmetics.
